# Supplementary material for: α11β1 Integrin is Induced in a Subset of Cancer-Associated Fibroblasts in Desmoplastic Tumor Stroma and Mediates In Vitro Cell Migration
Source: Cancers (Basel). 2019 Jun 1;11(6):765. doi: 10.3390/cancers11060765 (PMC6627481; doi:10.3390/cancers11060765)
Supplement: Supplementary file 1 [file cancers-11-00765-s001.pdf]

Supplementary Materials

α11β1 integrin is induced in a subset of cancer-associated fibroblasts in desmoplastic tumor stroma and mediates *in vitro* cell migration

Cédric Zeltz, Jahedul Alam, Hengshuo Liu, Pugazendhi M. Erusappan, Heinz Hoschuetzky, Anders Molven, Himalaya Parajuli, Edna Cukierman, Daniela-Elena Costea, Ning Lu and Donald Gullberg

Supplementary Figures S1-S3

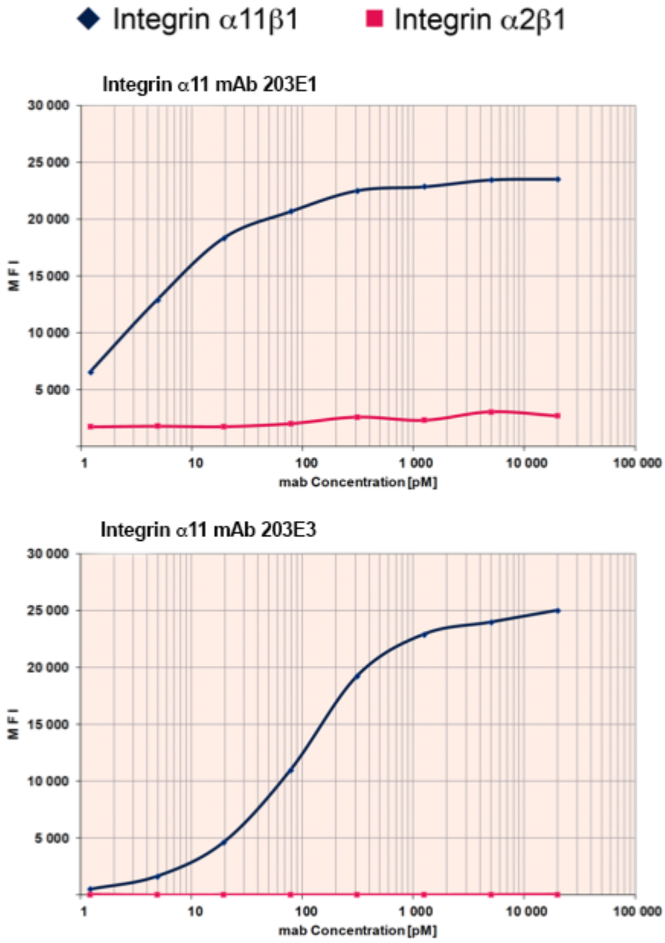

Figure S1. Determination of the integrin α11 203E1 and 203E3 mAb affinity. The experiment was done in nanoTools using Luminex beads (Biorad) conjugated with either integrin α11β1 or α2β1 protein (target protein, both are from R&D Systems). Binding affinity of the mAb 203E1 and 203E3 to the target protein was indicated by the mean fluorescent intensity (MFI) at different mAb concentrations.

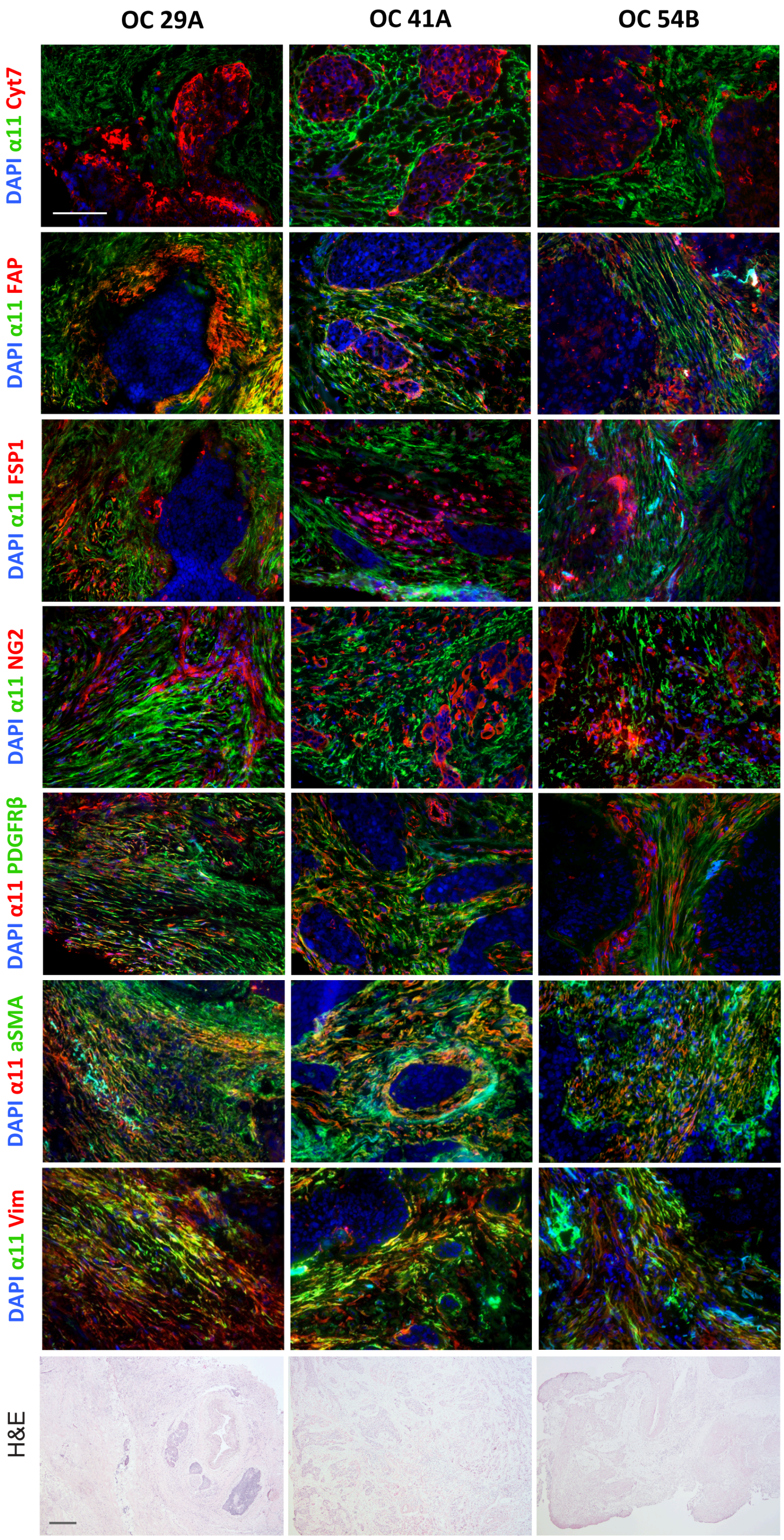

Figure S2. Immunostaining and H&E staining of sections from three different HNSCC patients. Immuno and H&E stainings were performed in available sections from 3 independent patients with an oral cancer (patient OC 29A, OC 41B and OC 54B). A representative staining result from OC 29A was shown in Figure 5 indicated as HNSCC, together with staining result from a PDAC patient. Scale bar: 100 µm in IF pictures and 200 µm in H&E pictures.

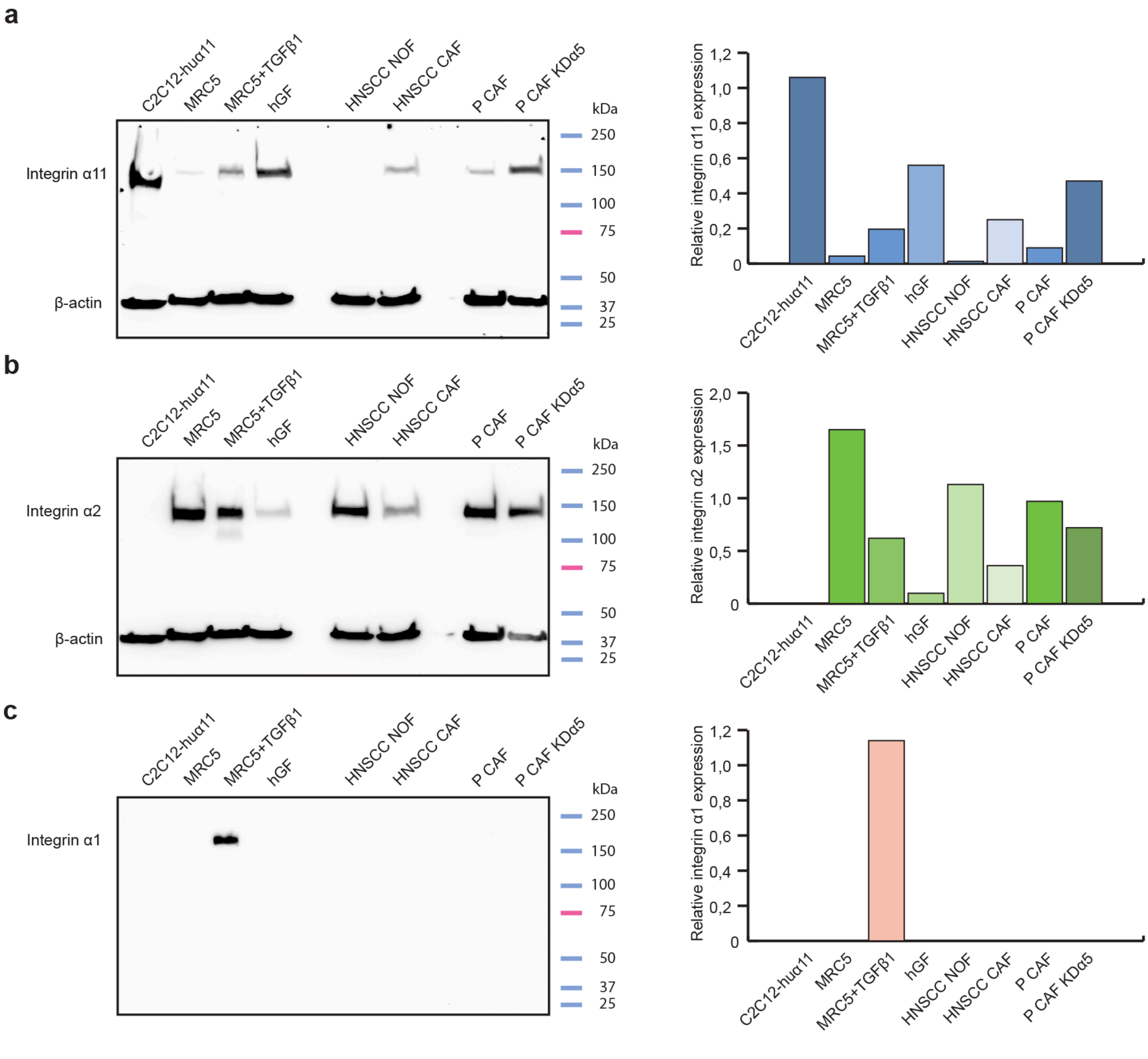

Figure S3. Full-size Western blots of Figure 6a and protein quantifications for each blot. Protein extracts from indicated cells were transferred to a PVDF membrane, and the membrane was blotted sequentially with antibodies to integrin α11 **(a)**, integrin α2 **(b)** and integrin α1 **(c)**. The protein-antibody complexes were stripped off before each blotting. Molecular weight marker (BioRad) was used and sizes of the bands were indicated. The relative expression levels of the integrin α chains were normalized to β-actin on each membrane except for integrin α1, in which the β-actin bands from integrin α2 blot were used since the integrin α1 blot was only incubated with α1 but not β-actin antibody.
